# Improving Clinical Management in ENT Casualty: A Multi-domain Quality Improvement Project Using a Digital Toolkit

**DOI:** 10.7759/cureus.95483

**Published:** 2025-10-27

**Authors:** Luke Borg, Ryan Gauci, Ryan Attard, Martine Grech, Mr Kenneth Muscat

**Affiliations:** 1 Department of Surgery, Mater Dei Hospital, Msida, MLT; 2 Department of Otorhinolaryngology, Mater Dei Hospital, Msida, MLT

**Keywords:** acute otitis externa, ent doctor, epistaxis, epistaxis management, nasal fracture, quality improvement tool, sudden sensorineural hearing loss

## Abstract

Background: Foundation year (FY) doctors rotating through otorhinolaryngology (ENT) are frequently required to manage high-risk acute presentations with limited speciality training, leading to variability in care quality and documentation.

Aim: This quality improvement (QI) project evaluated whether a digital clinical decision-support toolkit could improve adherence to evidence-based benchmarks across four common ENT casualty presentations: otitis externa, nasal fracture, sudden sensorineural hearing loss (SSNHL), and epistaxis.

Methods: Two audit cycles were performed over 10 weeks in a tertiary centre, including 203 patients in cycle 1 and 192 patients in cycle 2. Between cycles, a real-time ENT toolkit website with quick response (QR) code access, structured documentation templates, and guideline summaries was introduced to FY doctors at induction. Compliance with international standards was compared across cycles, supplemented by staff survey feedback using a five-point Likert scale.

Results: Significant improvements were observed in multiple domains. Documentation of tympanic membrane integrity in otitis externa increased from 34.3% to 67.7%. Timely manipulation under anaesthesia (MUA) for nasal fracture improved from 92.9% to 100%. Audiogram performance within 24 hours for SSNHL rose from 33.3% to 80%, and appropriate cautery use in epistaxis increased from 87.2% to 98.1%. Reductions in unnecessary imaging and swabbing were also achieved. Staff survey responses indicated improved confidence, efficiency, and guideline accessibility.

Conclusions: A low-cost, user-designed digital toolkit meaningfully improved clinical quality, documentation, and practitioner confidence in ENT casualty practice. This model demonstrates scalability across acute specialities where junior-led care is common.

## Introduction

Emergency ENT presentations constitute a substantial workload in secondary care, ranging from self-limiting otitis externa to potentially life-threatening haemorrhage. In many centres, frontline responsibility falls to foundation year (FY) doctors who often have limited prior ENT exposure. This mismatch between case complexity and practitioner experience has been associated with variability in documentation quality, suboptimal adherence to guidelines, and delayed escalation to senior staff.

Although clinical guidance from ENT UK, National Institute for Health and Care Excellence (NICE), the British Rhinological Society, and the American Academy of Otolaryngology-Head and Neck Surgery (AAO-HNS) is available, these resources are frequently underutilised in casualty settings due to accessibility barriers [1-6]. Previous work, including the INTEGRATE national ENT audit, has highlighted unwarranted variation in care and the need to embed evidence-based practice into everyday workflows [4].

Digital interventions such as quick response (QR) code-linked pathways and structured decision-support tools have shown promise in emergency and surgical specialities, improving compliance and practitioner confidence by delivering guidelines directly at the point of care [7-9]. However, to our knowledge, no multi-domain ENT casualty quality improvement (QI) project evaluating a real-time digital toolkit has been published.

This study aimed to measure improvements in documentation and management accuracy before and after implementation of a structured ENT toolkit, evaluating adherence to international benchmarks across four sentinel casualty presentations: otitis externa, nasal fracture, sudden sensorineural hearing loss (SSNHL), and epistaxis.

## Materials and methods

Design and setting

This was a two-cycle clinical audit conducted in the ENT casualty service of a tertiary referral hospital over 10 consecutive weeks (five weeks per cycle). The service routinely manages a high daily turnover of acute ENT cases, many of which are initially clerked by FY doctors. The audit was prospectively registered with the institutional Clinical Audit Committee and conducted in line with local clinical governance standards. Each cycle involved a different cohort of FY doctors due to scheduled rotation changeover, which has been acknowledged as a potential confounding factor.

Participants and inclusion criteria

Eligible cases included first-time attendances with one of four sentinel presentations: otitis externa, nasal fracture, SSNHL, or epistaxis. Patients attending for chronic disease follow-up, postoperative reviews, or where documentation was incomplete were excluded. Cycle 1 comprised 203 cases, while cycle 2 included 192 cases. Sampling included all consecutive eligible patients within the defined five-week period of each cycle.

Intervention: the ENT toolkit

Between audit cycles, the ENT toolkit was launched as a real-time, web-based decision-support platform. The toolkit was designed by junior doctors in consultation with senior ENT clinicians to ensure usability and clinical accuracy. It included QR-code access points displayed prominently in casualty and treatment rooms, enabling immediate mobile-device access.

The toolkit provided condition-specific management pathways based on international recommendations [1-6]. These pathways were supported by structured documentation templates covering history, examination, and escalation, as well as educational diagrams and checklists for practical procedures such as cautery and nasal packing. The toolkit was introduced to incoming FY doctors at induction, with a short training session to ensure familiarity.

Benchmark selection

Audit benchmarks were derived from national and international guidelines. For otitis externa, standards were taken from the NICE Clinical Knowledge Summaries [1]. For nasal fractures, benchmarks were drawn from the ENT UK guidance [2]. For SSNHL, criteria were defined using the ENT UK position statement [3] and AAO-HNS guidelines [6]. For epistaxis, reference was made to the British Rhinological Society guideline [4]. Additional standards were informed by the INTEGRATE national audit [4] and NICE NG98 on adult hearing loss [5].

Benchmarks were operationalised as measurable variables. For example, a timely audiogram in SSNHL was defined as completion within 24 hours of presentation, while an appropriate review of nasal fracture was defined as specialist assessment within seven days.

Data collection and analysis

A standardised proforma was used to capture demographics, management steps, and documentation quality for each case. Data were anonymised and entered into Microsoft Excel (Microsoft Corporation, Redmond, WA). Categorical variables were compared across cycles using chi-square testing, with p < 0.05 considered statistically significant. Given the small sample size in SSNHL, findings in this domain were interpreted descriptively.

Staff confidence and satisfaction were assessed through a five-point Likert scale survey administered before and after toolkit implementation. The Likert scale, originally described by Likert (1932), is in the public domain and free for use [10]. Scores were averaged and supplemented by free-text responses. A total of 35 FY doctors (18 pre-intervention, 17 post-intervention) completed the survey.

## Results

**Figure 1:**
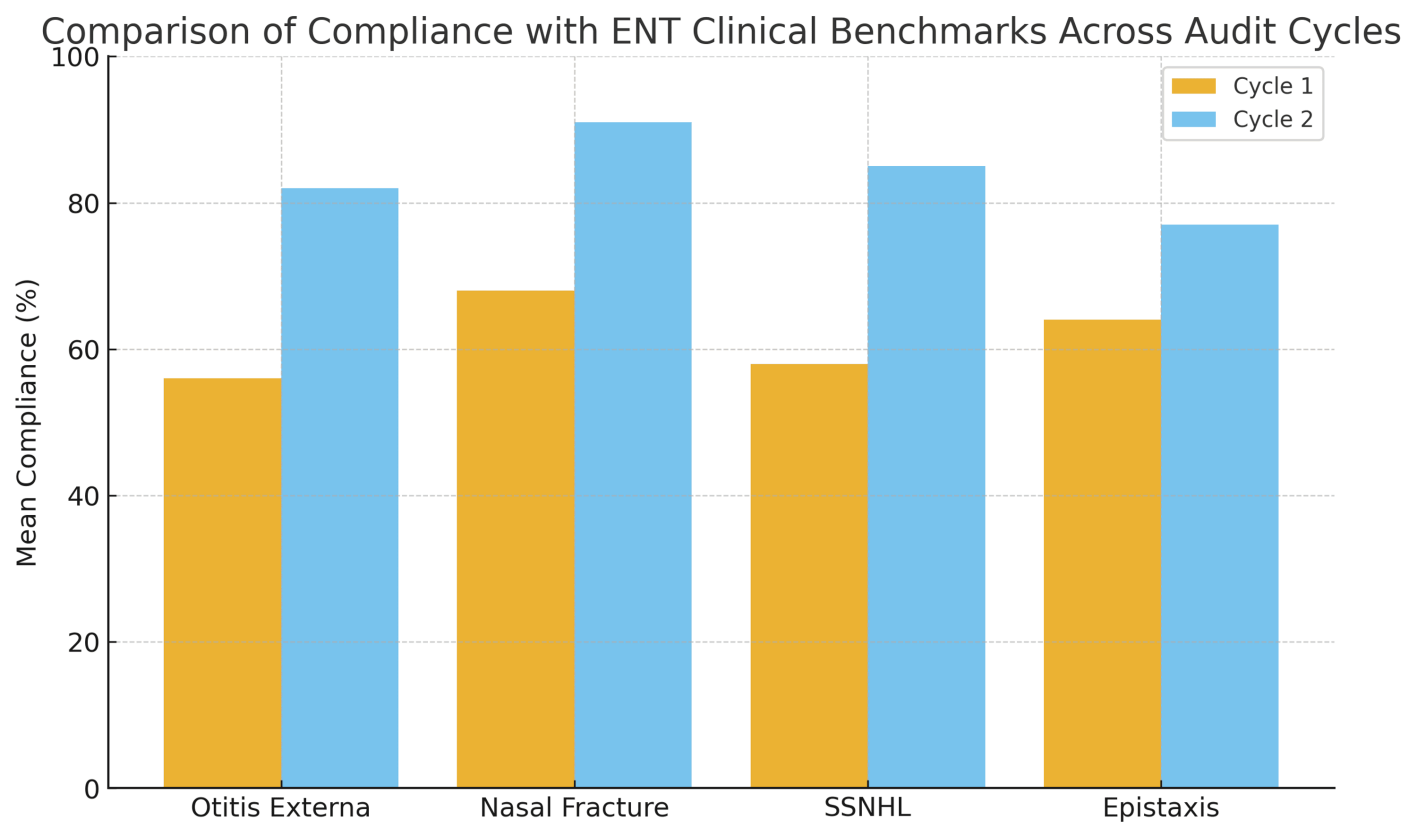
Comparison of mean compliance with international clinical benchmarks for otitis externa, nasal fracture, sudden sensorineural hearing loss (SSNHL), and epistaxis across two audit cycles. Cycle 1 reflects baseline performance, while cycle 2 followed the introduction of the ENT toolkit.

Improvements were observed across all four domains. Full results are presented in Figure 1 (collective), Table 1 (otitis externa), Table 2 (nasal fracture), Table 3 (SSNHL), and Table 4 (epistaxis).

**Table 1:** Otitis externa management across two audit cycles. Comparison of adherence to the National Institute for Health and Care Excellence (NICE) Clinical Knowledge Summaries: Otitis externa [1] benchmarks before and after implementation of the ENT toolkit.

Otitis externa	Cycle 1 (n = 65)	Cycle 2 (n = 72)
Prescription of topical antibiotic therapy	76.9%	82.3%
Tympanic membrane integrity documented	34.3%	67.7%
Ear wick is used when the canal is occluded	80.0%	90.6%
Avoidance of unnecessary ear swab	74.1%	84.4%
Documentation of water precautions	64.8%	83.3%

**Table 2:** Nasal fracture management across two audit cycles. Comparison of adherence to ENT UK nasal trauma guidelines [2] benchmarks before and after implementation of the ENT toolkit.

Nasal fracture	Cycle 1 (n = 24)	Cycle 2 (n = 19)
Reviewed within 7 days	58.3%	94.7%
Manipulation under anaesthesia (MUA) within 14 days	92.9%	100%
Assessment for septal haematoma documented	79.2%	84.2%
Avoidance of unnecessary imaging	41.7%	84.2%

**Table 3:** Sudden sensorineural hearing loss (SSNHL) management across two audit cycles. Comparison of adherence to the ENT UK position statement [3] and the AAO-HNS clinical practice guideline [6] benchmarks before and after implementation of the ENT toolkit.

Sudden sensorineural hearing loss	Cycle 1 (n = 6)	Cycle 2 (n = 5)
Audiogram performed within 24 hours	33.3%	80.0%
Steroid therapy prescribed	50.0%	100%
Urgent ENT referral made	66.7%	100%
MRI scan requested	83.3%	60.0%

**Table 4:** Epistaxis management across two audit cycles. Comparison of adherence to the British Rhinological Society epistaxis guideline [4] benchmarks before and after implementation of the ENT toolkit.

Epistaxis	Cycle 1 (n = 108)	Cycle 2 (n = 96)
Cautery attempted for a visible vessel	87.2%	98.1%
Nasal packing required	96.0%	78.4%
Senior escalation for posterior/refractory bleeds	78.0%	100%
Routine coagulation screen is avoided unless indicated	34.0%	61.0%
Documentation of patient counselling	28.0%	46.0%

Otitis externa

Documentation of tympanic membrane integrity improved significantly from 34.3% to 67.7% (p = 0.002), and avoidance of unnecessary ear swabs improved from 74.1% to 84.4% (p = 0.04). Water precautions were documented more consistently (64.8% → 83.3%, p = 0.01), consistent with the NICE guidance [1].

Nasal fracture

Review within seven days increased from 58.3% to 94.7% (p = 0.006), and manipulation under anaesthesia (MUA) within 14 days improved from 92.9% to 100% (p = 0.31). Avoidance of unnecessary imaging rose markedly from 41.7% to 84.2% (p = 0.005).

Sudden sensorineural hearing loss (SSNHL)

Timely audiogram performance improved from 33.3% to 80.0% (p = 0.09), steroid therapy increased from 50.0% to 100%, and urgent ENT referral improved to 100%. MRI requests decreased from 83.3% to 60.0%, indicating more selective use. These results, while not statistically significant due to small sample size (n = 6 and n = 5), are clinically relevant [3,6].

Epistaxis

Appropriate cautery for visible vessels increased from 87.2% to 98.1% (p = 0.03), while nasal packing rates fell from 96.0% to 78.4% (p = 0.008). Senior escalation improved from 78.0% to 100% (p = 0.02), and avoidance of unnecessary coagulation screening rose from 34.0% to 61.0% (p = 0.01).

Staff feedback

Survey results demonstrated improvements in confidence (2.4/5 → 4.1/5, p < 0.01), perceived accessibility of guidelines (2.1/5 → 4.5/5, p < 0.01), and satisfaction with documentation templates (2.8/5 → 4.3/5, p < 0.01). Free-text responses indicated perceived gains in efficiency, confidence, and patient safety.

Timely audiogram performance improved (33.3% → 80%), steroid initiation rose to 100%, and urgent referral increased to 100%. MRI requests decreased from 83.3% to 60%, reflecting more selective use but requiring careful alignment with the AAO-HNS recommendations [6].

## Discussion

This QI project demonstrates that embedding a digital decision-support toolkit into an ENT casualty environment can meaningfully improve adherence to evidence-based care, documentation quality, and practitioner confidence.

The improvements observed mirror findings in other specialities where digital pathways, checklists, and decision-support systems have enhanced clinical standardisation [7-9,11]. For example, sepsis bundles delivered through electronic prompts have improved time-to-antibiotic administration [8], while surgical safety checklists significantly reduced perioperative morbidity and mortality worldwide [11]. Our project shows that similar benefits can be achieved in ENT casualty practice.

A key strength was the focus on FY doctors, who often feel underprepared for ENT emergencies [12]. By providing QR-code-linked guidance and structured templates, the toolkit reduced reliance on memory and improved documentation quality. The reduction in unnecessary investigations, such as ear swabs and imaging, enhanced cost-effectiveness and antimicrobial stewardship [13]. Improved SSNHL management is especially relevant given the prognostic importance of timely steroids and audiogram assessment [3,6].

However, limitations must be acknowledged. This was a single-centre study with modest case numbers, particularly for SSNHL. The two audit cycles involved different FY cohorts, which may have influenced results. Additionally, awareness of the re-audit may have contributed to performance improvement (Hawthorne effect). Improvements were measured via documentation review rather than direct observation, introducing possible bias. Survey data were subjective and small in scale (n = 35). Long-term patient outcomes, such as complication or satisfaction rates, were not assessed.

Future directions include multi-centre trials, integration into electronic health records, and incorporation of real-time analytics. Expansion to paediatric ENT conditions and the addition of multimedia procedure guides could further enhance usability and sustainability.

## Conclusions

The introduction of a structured digital ENT toolkit improved compliance with clinical benchmarks, reduced unnecessary investigations, and enhanced practitioner confidence among FY doctors managing acute ENT cases. These findings highlight the potential of low-cost, user-designed digital interventions to address the gap between complex emergency presentations and limited junior doctor experience.

Given its feasibility, scalability, and positive reception among staff, this model may be adapted for use in other acute specialities where frontline care is often junior-led. Wider adoption and integration into institutional systems could drive meaningful improvements in quality, safety, and standardisation of emergency care delivery.

## References

[1] (2022). National Institute for Health and Care Excellence (NICE). Clinical knowledge summaries: otitis externa. https://cks.nice.org.uk/topics/otitis-externa/.

[2] (2011). ENT UK. Nasal trauma guidelines. https://www.entuk.org/_userfiles/pages/files/guidelines/global%20ent%20guidelines/nasal_trauma.pdf.

[3] (2020). ENT UK. Management of suspected unilateral idiopathic sudden sensorineural hearing loss in adults. https://www.entuk.org/resources/98/management_of_suspected_unilateral_idiopathic_sudden_sensorineural_hearing_loss_in_adults/.

[4] National ENT Trainee Research Network (2017). The British Rhinological Society multidisciplinary consensus recommendations on the hospital management of epistaxis. J Laryngol Otol.

[5] (2018). National Institute for Health and Care Excellence (NICE). Hearing loss in adults: assessment and management. NICE guideline NG98. https://www.nice.org.uk/guidance/ng98.

[6] Stachler RJ, Chandrasekhar SS, Archer SM (2012). Clinical practice guideline: sudden hearing loss. Otolaryngol Head Neck Surg.

[7] Campbell H, Hotchkiss R, Bradshaw N, Porteous M (1998). Integrated care pathways. BMJ.

[8] Levy MM, Evans LE, Rhodes A (2018). The Surviving Sepsis Campaign bundle: 2018 update. Intensive Care Med.

[9] Grygorian A, Montano D, Shojaa M, Ferencak M, Schmitz N (2024). Digital health interventions and patient safety in abdominal surgery: a systematic review and meta-analysis. JAMA Netw Open.

[10] Likert R (1932). A technique for the measurement of attitudes. Arch Psychol.

[11] Haynes AB, Weiser TG, Berry WR (2009). A surgical safety checklist to reduce morbidity and mortality in a global population. N Engl J Med.

[12] Hannaford PC, Simpson JA, Bisset AF, Davis A, McKerrow W, Mills R (2005). The prevalence of ear, nose and throat problems in the community: results from a national cross-sectional postal survey in Scotland. Fam Pract.

[13] Little P, Stuart B, Hobbs FD (2014). Antibiotic prescription strategies for acute sore throat: a prospective observational cohort study. Lancet Infect Dis.

